# Greedy Firefly Algorithm for Optimizing Job Scheduling in IoT Grid Computing

**DOI:** 10.3390/s22030850

**Published:** 2022-01-23

**Authors:** Adil Yousif, Samar M. Alqhtani, Mohammed Bakri Bashir, Awad Ali, Rafik Hamza, Alzubair Hassan, Tawfeeg Mohmmed Tawfeeg

**Affiliations:** 1 Department of Computer Science, College of Science and Arts-Sharourah, Najran University, Sharourah 68341, Saudi Arabia; aaomar@nu.edu.sa; 2 Department of Information Systems, College of Computer Science and Information Systems, Najran University, Najran 61441, Saudi Arabia; smalqhtani@nu.edu.sa; 3 Department of Math, Turubah University College, Taif University, Taif 26571, Saudi Arabia; safi@tu.edu.sa; 4 Department of Computer Science, Faculty of Computer Science and Information Technology, Shendi University, Shendi 41601, Sudan; 5 National Institute of Information and Communications Technology, Tokyo 184-8795, Japan; rafik.hamza@nict.go.jp; 6 Department of Computer Science, School of Computer Science and Informatics, University College Dublin, Belfield, D04 V1W8 Dublin, Ireland; alzubair.mohamedtahir@ucd.ie; 7 Lero-the Irish Software Research Centre, Tierney Building, University of Limerick, Sreelane, V94 NYD3 Limerick, Ireland; 8 Department of Computer Science, Faculty of Computer Science and Information Technology, University of Science and Technology, Khartoum 14411, Sudan; tawfeeg.mohammed@ust.edu.sd

**Keywords:** grid, IoT, job scheduling, greedy, firefly algorithm

## Abstract

The Internet of Things (IoT) is defined as interconnected digital and mechanical devices with intelligent and interactive data transmission features over a defined network. The ability of the IoT to collect, analyze and mine data into information and knowledge motivates the integration of IoT with grid and cloud computing. New job scheduling techniques are crucial for the effective integration and management of IoT with grid computing as they provide optimal computational solutions. The computational grid is a modern technology that enables distributed computing to take advantage of a organization’s resources in order to handle complex computational problems. However, the scheduling process is considered an NP-hard problem due to the heterogeneity of resources and management systems in the IoT grid. This paper proposed a Greedy Firefly Algorithm (GFA) for jobs scheduling in the grid environment. In the proposed greedy firefly algorithm, a greedy method is utilized as a local search mechanism to enhance the rate of convergence and efficiency of schedules produced by the standard firefly algorithm. Several experiments were conducted using the GridSim toolkit to evaluate the proposed greedy firefly algorithm’s performance. The study measured several sizes of real grid computing workload traces, starting with lightweight traces with only 500 jobs, then typical with 3000 to 7000 jobs, and finally heavy load containing 8000 to 10,000 jobs. The experiment results revealed that the greedy firefly algorithm could insignificantly reduce the makespan makespan and execution times of the IoT grid scheduling process as compared to other evaluated scheduling methods. Furthermore, the proposed greedy firefly algorithm converges on large search spacefaster , making it suitable for large-scale IoT grid environments.

## 1. Introduction

The computational grid is a distributed technology consisting of multiple distributed heterogeneous resources in different network sites [1,2,3]. The Internet of Things (IoT) is defined as interconnected digital and mechanical devices with intelligent and interactive data transmission features over a defined network [4,5]. Grid computing is a large-scale computational environment consisting of a huge number of heterogeneous resources combined to perform complex processes [6]. From a communication viewpoint, the IoT is identified as a system of interconnected things, each with a unique address to deliver computational services and connect using specified protocols. The IoT comprises things such as sensors, computing devices, embedded systems, objects, and animals with unique identifiers and that have the capacity to transmit data over a specified network [4,5,7,8,9]. The IoT grid was introduced to integrate the grid services with IoT architecture [10,11]. The IoT grid benefits from deploying grid service architecture for IoT technology [10,12]. The grid sites are clusters, super computers, or multiprocessor computing networks [1]. Moreover, global resources and tasks send application jobs through the global grid job scheduler. Given several clients’ jobs and several heterogeneous resources, the grid job scheduling problem is finding a schedule that maps the client’s jobs to site resources to optimize specified scheduling criteria [13,14]. The complexity of the computational grid increased due to the high heterogeneity of grid resources, the complexity of computational problems, and the dynamic nature of resources [15]. These complexities of computational grid environments offer an opportunity and challenge for developing grid job scheduling mechanisms based on metaheuristics and nature-inspired methods to optimize the grid job scheduling process. The job scheduling process aims to find the optimal mapping of clients’ jobs to grid resources that minimize the execution and makespan times [16]. The exponential increase in the size of the solution search space inspired more research to utilize optimization methods to tackle grid job scheduling gaps [17].

Nature-inspired metaheuristics have established a high degree of success in solving optimization problems with large scale search space [18,19]. Several genetic algorithm (GA) methods have been introduced for grid job scheduling problems [20,21,22]. To improve the genetic algorithm further, a hybrid job clustering combining fuzzy C-Mean and a genetic algorithm was developed in [23]. A task scheduler with fault tolerance based on ACO is proposed to guarantee that tasks are performed efficiently, even if resource failure has arisen [24].

The firefly algorithm is a nature-inspired metaheuristic based on the flashing behavior of fireflies [25]. A study in [26] presented job scheduling mechanisms for grid computing based on the standard firefly algorithm. Each firefly algorithm denotes one schedule in the solution search space in the standard firefly algorithm. The job scheduling standard mechanism based on FA starts with an initial random population of schedules or fireflies [26]. In  each stage, greedy algorithms rely on current options to make their decisions and do not consider future possibilities [27,28].

This paper proposed a greedy firefly algorithm (GFA) for IoT grid job scheduling. The greedy firefly algorithm enhances the standard firefly algorithm by combining the firefly method with the greedy mechanism. In the proposed greedy firefly algorithm, the greedy method is utilized as a local search mechanism to enhance the speed of convergence and efficiency of the solution generated by the standard firefly algorithm. The proposed GFA aims to obtain better coverage as the search space increases. This is because the greedy algorithm increases the search exploitation process. The search exploitation implemented by the greedy algorithm increases the probability of finding the optimum solution. Furthermore, the study considers the balance between exploitation achieved by the greedy algorithm and the exploration implemented by the firefly algorithm.

The remainder of this paper is organized as follows. Section 2 reviews the related works. Section 3 discusses the basic idea of the proposed greedy firefly algorithm. Section 4 describes experiments and results. Finally, we conclude in Section 5.

## 2. Related Works

The IoT grid was introduced to integrate the grid services with IoT architecture [10]. The IoT grid benefits from deploying grid service architecture to IoT technology [10,12]. Several researchers have presented hybrid high-performance computing systems with IoT technologies [29,30,31]. The research by Deniziak and Bąk in [32] suggests a new scheduling mechanism for IoT distributed jobs in heterogeneous cloud computing. The researchers considered that jobs that demand execution are identified as directed acyclic graphs. According to [33], grid computing is a group of clusters connected over high-speed networks that involve coordinating and sharing computational power, data storage, and network resources operating across dynamic and geographically dispersed locations. In the desktop grid model, the job is submitted for execution only when the system is idle, and there is no guarantee that the job will entirely execute without disruption. In 2017, Shiny and Jetlin described a secure resource allocation method capable of allocating the resources to authenticated grid users by improving the system’s functionality by submitting the jobs on a machine with a higher probability of being available at a given time [33]. Furthermore, they proposed a system where the number of jobs was fewer than the available resources, with checkpointing and replication tools were being used to mitigate the volatility of the resource, hence reducing job processing time. In the same year, an improved ant colony optimization algorithm with fault tolerance for job scheduling in the grid computing system was proposed to ensure that jobs were executed successfully even when resource failure occurred. In addition, the design applied a resource failure and checkpoint-based rollback recovery strategy to reduce the amount of work lost upon system failure by immediately saving the system’s state [24].

An Application-aware Deadline Constraint Job Scheduling Mechanism On A Large-Scale Computational Grid was proposed to overcome earlier models’ challenges due to the heterogeneity, dynamics of resources, and diversity of application requirements [34]. Since the scheduling task had remained a challenging task, a Hybrid Heuristic of Variable Neighborhood Descent and Great Deluge Algorithm for efficient task scheduling in grid computing was proposed. It synergized the Great Deluge algorithm and variable Neighborhood Decent algorithm to schedule independent tasks, effectively minimizing the makespan [35]. Furthermore, a fuzzy priority deadline-based task scheduling algorithm (FPDSA) having a fuzzy deadline limitation to competent job execution was proposed in 2020 to enhance the existing grid’s performance regarding Average Actual execution and the number of non-delayed Jobs [15]. A recent survey showed that most of the algorithms developed a long time ago are in constant development, making them still used today due to their efficiency. These algorithms include the Ant Colony algorithm, Compact Genetic algorithm, Load Balanced Min-Min algorithm, and Divisible Load Scheduling algorithm. They are still widely used for scientific and medical purposes [36]. To guarantee that jobs complete their execution within the estimated completion time, a dynamic job scheduling model (DTSCA) that uses job characteristics to map them to resources with minimum execution time was proposed. The system would make virtual machine choices closer to their expectations [37]. The recent development has been tailored to provide a very low-cost distributed computing platform to group members using their personal computers. The proposed Static Job Scheduling Algorithm Considering CPU Core Utilization for User-PC Computing System has the master PC receiving user tasks and assigning them to available worker PCs [38]. High-Performance Computing (HPC) systems offer massive computation strength to execute large-scale applications, hence consuming a lot of energy and increasing carbon emissions. To overcome these drawbacks and improve searching efficiency, a performance-aware, energy-efficient parallel job schedule in HPC grids using nature-inspired hybrid meta-heuristics was proposed [1].

Nature-inspired optimization methods have been applied to handle the job scheduling problem on the computational grid [39]. Natural metaheuristics are derived and inspired by natural behaviors to address complex real-world issues [40]. The essential methods representing the field are evolutionary optimization techniques and swarm intelligence mechanisms [28,41]. Metaheuristic evolutionary techniques such as Genetic Algorithms (GA) and Differential Evolution (DE) methods have proven their usefulness in solving the problem of spanned design space [41,42]. In recent years, swarm intelligence (SI) mechanisms such as PSO and FA have been considered successful search methods that perform better than evolutionary techniques when applied to various problems [39].

GA starts with a random population of candidate solutions. The initial random population is a set of integer numbers, and each solution in the population denotes a chromosome. To enhance GA further, a hybrid job clustering using a fuzzy C-Mean and a genetic algorithm was developed in [23]. This hybrid genetic algorithm was presented to reduce the GA’s generations’ repetitions. Delavar at el. [43] presented a job scheduling method to attain a shorter execution times and lower communication costs. A rank-based genetic for the computational grid is introduced to increase the genetic algorithm convergence and reduce the search time [44]. A new research work presented two hybrid meta-heuristic job scheduling methods for the computational grid.

A task schedule with fault tolerance based on ACO is proposed to guarantee that tasks are performed efficiently even if resource failure has arisen. The ACO fault tolerance task scheduling mechanism is based on the resource failure rate and the checkpoint-rollback approach. The check-pointing parts of the mechanism aim to decrease the total effort lost during grid failure by instantly saving the status of the grid computing system [24]. A grid architecture for scalable monitoring and enhanced, dependable job scheduling is presented in [45]. The proposed scalable monitor architecture focuses on two critical distributed heterogeneous and multi-domain grid computing features: the scalable distribution of control and administration data and system recovery of when job failures occur [45]. On the other hand, HC is used in grid task scheduling [46,47]. HC optimization has a plateau problem in flat search space. In the plateau problem, HC can not find the next best position. HC will occasionally choose directions that do not have the best schedules [48]. Tabu Search (TS) has an advantage over HC as it has a memory that supports continuous exploration, even if the mechanism does not generate better solutions. In addition, the memory avoids TS searching from local optima trapping.

The basic job scheduling approaches, such as shortest job first, select the best schedule by considering a single criterion. For instance, the goal of MET is to map tasks with their most suitable resources. Still, this can produce a serious load imbalance within resources. Similarly, the FCFS mechanism may produce weak scheduling in a task with high resource requests being submitted to the global scheduler, causing long execution times for several grid resources [15].

The greedy approach Tabu Search (TS) utilizes a one-search path of job scheduling. This constraint generates scheduling solutions appropriate for lightweight scheduling problems. Yet, it is challenging for the single path approaches to obtain the optimal schedules in case of heavy system loads. Furthermore, single path scheduling mechanisms suffer from plateau problems and solution search space diversity issues. As a result of these weaknesses, TS generates long execution times in heavy load system states.

Genetic algorithms and other evolutionary algorithms are sometimes trapped in local optimals. As a result, they are unable to progress any further extent [49]. This happens as the diversity of schedules in the population is decreased, which makes the crossover and mutation processes not capable of producing enhanced chromosomes [50]. Likewise, evolutionary algorithms sometimes suffer in obtaining optimal schedules due to the difficulty in handling the population changes [51]. The limitations of evolutionary algorithms influence the job scheduling process on the computational grid as GA, and DE generate long makespan and flowtime in some scenarios [50,52,53,54,55].

Swarm intelligence job scheduling approaches such as PSO and ACO suffer from several problems. For instance, PSO reduces the convergence speed while close to the optimal schedule. In addition, PSO scheduling solutions need to handle partial optimism issues. Even if scheduling search space convergence is secured in ACO, the time to convergence is still uncertain [56].

The study [57] presented a method using ant-based systems to reproduce and represent grid service information on the computational grid, allowing a specific semantic classification of such facilities. System information is distributed using agents, which navigate the computational grid using P2P interconnections between grid sites.

## 3. The Proposed GFA for IoT Grid Job Scheduling

**Figure 1:**
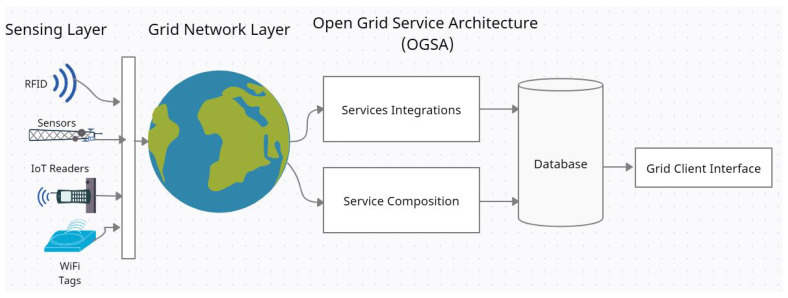
IoT Grid Environment.

This paper proposed an enhanced greedy firefly algorithm for job scheduling on the IoT grid. The proposed greedy algorithm is based on a discrete firefly algorithm. The study applied the smallest position value (SMV) mechanism [58]. The idea behind the proposed greedy firefly algorithm is to combine the benefits of the firefly algorithm and greedy algorithms by using the greedy choice to help enhance the firefly algorithm schedule. Furthermore, the greedy choice allows the firefly solution to search for schedules’ near-optimal solutions. The IoT grid environment is presented in Figure 1. The IoT grid environments consist of four layers: a sensing layer, a grid network layer, an open grid service architecture layer, and the client interface layer. The sensing layer contains the sensors’ RFID readers and other IoT devices. The grid network layer represents the grid computing network core. The third layer is an open grid service architecture in which service integration is provided between grid resources and IoT devices. The last layer is the IoT grid user interface. The process starts when grid clients build an IoT application that needs computational grid power. Next, the client application is submitted to grid resources, and clients can require additional resource specifications. When the execution process starts, the information requested by the sensors is collected using the embedded system’s devices. The next step includes submitting the execution request to the grid IoT scheduler. The grid IoT scheduler schedules all the submitted jobs based on the proposed scheduling mechanism. Finally, the execution results are sent back to the IoT system.

**Figure 2:**
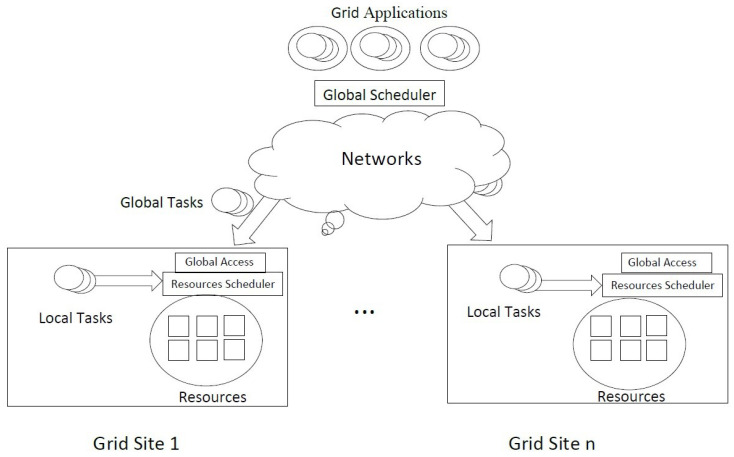
GFA IoT Grid Task Scheduling Architecture.

Figure 2 depicts the elements and components of IoT grid scheduling architecture. The IoT grid applications send their tasks to the global grid scheduler. The proposed greedy firefly algorithm-based global scheduler distributes IoT application tasks to the appropriate resources in the available sites. Each grid site has its local tasks and receives tasks from other sites using the global scheduler. The IoT grid applications started submitting jobs to the global scheduler. Then, the global scheduler submits jobs to suitable IoT grid resources using the specific scheduling method using the network connections. Each grid site consists of several resources, a local scheduler, and a global access component. The local scheduler is responsible for the local scheduling policy. The global access components allow the global scheduler to access the local resource information. Finally, the proposed GFA is performed within the global scheduler component.

### 3.1. Mathematical Modeling

In this study, the following assumptions are considered:

- All clients’ jobs are independent.
- The submitted jobs have different executions times.
- The model assumes that the IoT grid resources deliver only one type of service.
- Pre-emption is not allowed, i.e., each job cannot be interrupted before its completion on the assigned resource.
- At any given time, a resource can run only one job.

**Figure 3:**
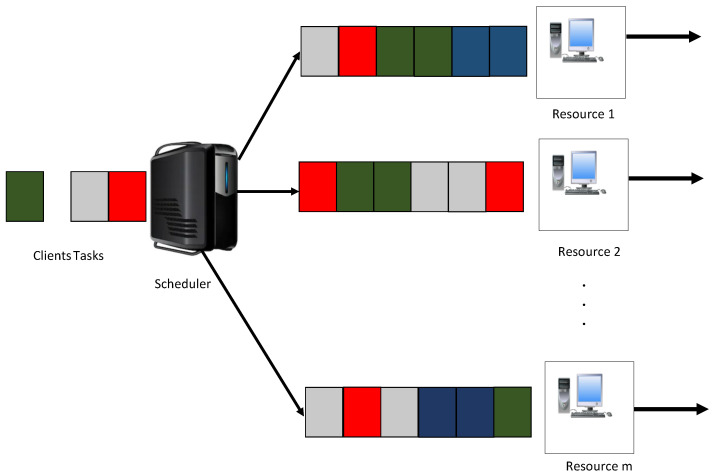
Model of IoT Grid Job Scheduler.

To formulate the IoT grid job problem, this study consider n clients jobs $J_{n}$ = $\left[j_{1},j_{2},j_{3},\ldots ,j_{n}\right]$ and m grid resources $R_{m}=\left[r_{1},r_{2},r_{3},\ldots ,r_{m}\right]$ with an objective of minimizing job execution and makespan times. The speed of resources is measured in million instructions per second (MIPS). The job length is defined as the number of instructions in millions (MI). Figure 3 describes the IoT grid job scheduler model. Jobs allocated to each resource are handled on a first-come, first-serve basis. If a resource is busy, jobs are queued in the resource queue.

**Figure 4:**
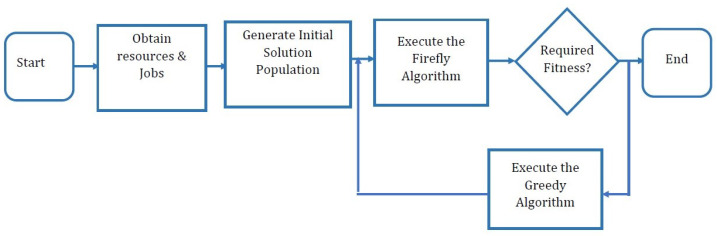
The Flowchart of the Proposed GFA.

This study considers the execution and makespan times to be minimized. First, the study defined $C_{j}$ as the time that the last job, $j_{k}$ completed execution. Consider $C_{max}=max\left\{C_{j},j=1,\cdots ,n\right\}$ as representing makespan time and $\sum_{j=n}^{j=1}c_{j}$ represents the execution time. The proposed scheduling method improves the standard firefly algorithm presented in [25]. In the proposed method, the study combines the standard firefly algorithm with the greedy algorithm to enhance the scheduling and minimize the makespan time. In the greedy firefly algorithm, the greedy method is employed as a local search mechanism to improve the rate of convergence and quality of the solution generated by the standard firefly algorithm, as shown in Figure 4.

The proposed GFA starts when grid clients submit jobs to the global scheduler. After that, the global scheduler gathers information of resources in each grid site. After obtaining jobs’ and resources’ information, the proposed GFA generates a random population of solutions from the solution search space. In each population, the solution represents a firefly. The makespan time and execution times are considered as firefly attractiveness. Starting with the random initial solutions, the firefly algorithm is executed. If the new population achieves the required fitness value, the algorithm finishes. However, suppose the new population does not produce the required fitness. A greedy algorithm is then implemented to exploit the solution search space by searching for better solutions near the current solution. This is achieved by searching and moving to solutions near the existing solutions. The algorithm then returns to the firefly algorithm execution. This process is repeated until the required fitness value is achieved.

The continuous firefly algorithm needs to be modified to solve discrete problems such as job scheduling problems. Discrete optimization problems apply modified nature-inspired metaheuristics optimization methods [59]. The study used the smallest position value rule (SPV) [58]. Several studies utilized the SPV rule to adjust the continuous optimization with a discrete computational problem [58,60,61,62].

### 3.2. Greedy Algorithm

The greedy method is integrated with the standard firefly algorithm as a local search mechanism to enhance the speed of convergence and efficiency of the solution generated by the standard firefly algorithm. The idea of greedy scheduling is that there is a group of jobs to be scheduled on some resources, and each job *j* has a given length $l_{j}$. The greedy scheduling method aims to schedule as many jobs as possible on the cloud provider’s resources. Starting from the empty schedule, provided at least one cloud job exists, the jobs that lead to minimum makespan time are continuously added. A The greedy algorithm can choose any option that may seem to be the best in a particular step, and then it resolves partial problems that will appear later. In each phase, the greedy algorithm selects a solution, and this selection process is based only on the current phase and not the future phases. The greedy algorithm repeats the generations of a greedy solutions to transform a given problem into a smaller one. Sometimes, greedy algorithms fail to obtain the best solution. Furthermore, the greedy algorithm can generate the worst solution from the search space. The details of the proposed Greedy Algorithm for the IoT grid job scheduling steps are stated in Algorithm 1.
**Algorithm 1** Greedy Algorithm for Job Scheduling.**Require:** $Greedy_{S}chedule\left(s,f\right)$1:$J\leftarrow submitted_{J}obs$2:R←available_Resources3:S←empty4:$0\leftarrow f_{makspan}$5:Sort jobs *J* by increasing order of length6:**while**(iteration_bestiteration<nmax)**do**7:    iteration=iteration+18:    **for** (job=1) to *n* **do**9:        **for** (resource=1) to *m* **do**10:           assign $job_{i}=resource_{j}$11:           $C_{i,j}\leftarrow finish\_time$12:           **if** $C_{i,j}\leftarrow prev\_finish$ **then**13:               $C_{i,j}>prev\_finish$14:               $C_{i,j}\leftarrow prev\_finish$15:               $\sum_{j=1}^{n}C_{i,j}\leftarrow T_{i}$16:           **end if**17:        **end for**18:        $max\left(T_{i}\right)\leftarrow f_{makspan}\left(c\right)$19:        S∪(j)←S20:    **end for**21:    return *S*22:**end while**

### 3.3. Firefly Algorithm

Firefly algorithm is a nature-inspired optimization. Each firefly attracts mating partners and preys on other fireflies using flashing lights [39,63,64]. The details of the proposed firefly algorithms for IoT grid job scheduling steps are stated in Algorithm 2.
**Algorithm 2** Firefly Algorithm for Job Scheduling.**Require:** Form each firefly to represent a schedule1:max←maxIteration2:generate initial random population of fireflies3:Sort jobs *J* by increasing order of length4:**while**(iteration<max)**do**5:    **for** each firefly **do**6:        calculate the light intensity as makespan time7:        estimate the firefly’s distances8:        estimate the firefly’s attractiveness9:        **if** the firefly has the highest brightness value **then**10:           the firefly has the highest brightness value11:        **else**12:           **if** the firefly has the highest brightness value **then**13:               execute the greedy algorithm14:               move the firefly to the firefly with highest brightness value15:           **end if**16:        **end if**17:    **end for**18:    Return the best firefly19:**end while**

**Figure 5:**
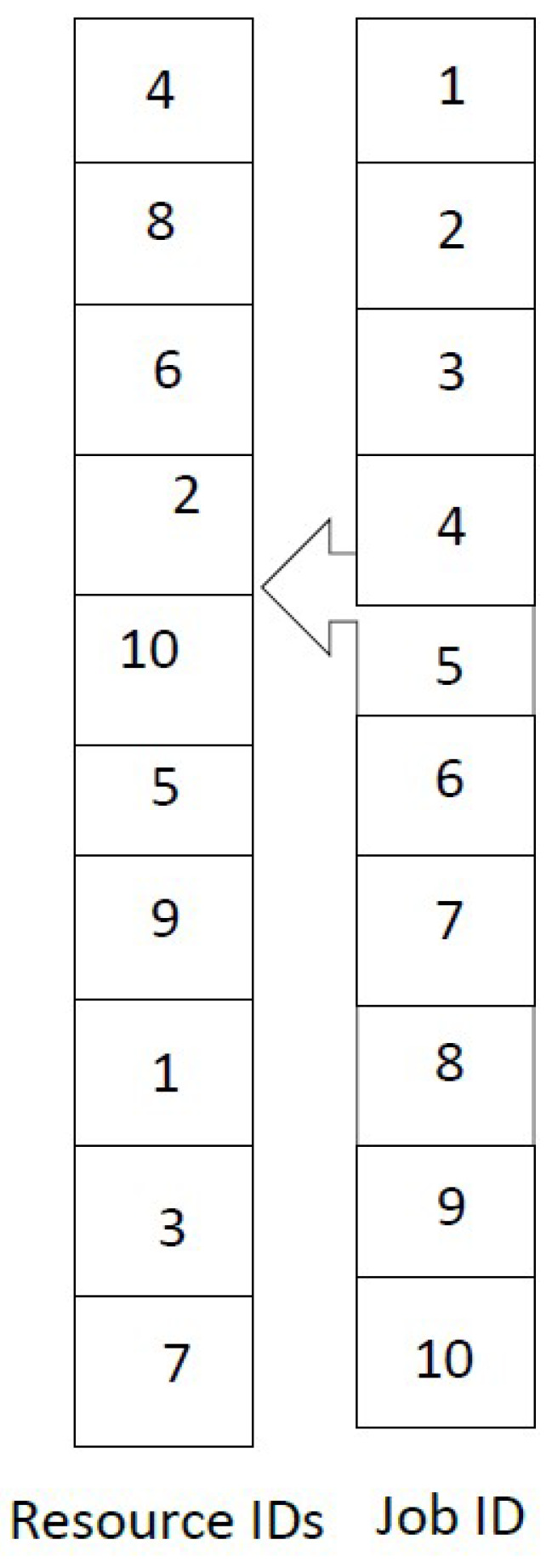
The Firefly Permutation Representation of a Valid Schedule.

Figure 5 describes an example of a valid firefly algorithm schedule. As shown in Figure 5, job number 1 is scheduled to the resource with ID 4, and job number 2 is scheduled to resource with ID 8, and so on.

### 3.4. The Proposed GFA in Details

This section explains the proposed job scheduling mechanisms using the greedy firefly algorithm. To illustrate the proposed greedy firefly algorithm clearly, an example instance with nine jobs and four resources are defined in Table 1 and Table 2, respectively.

**Figure 6:**
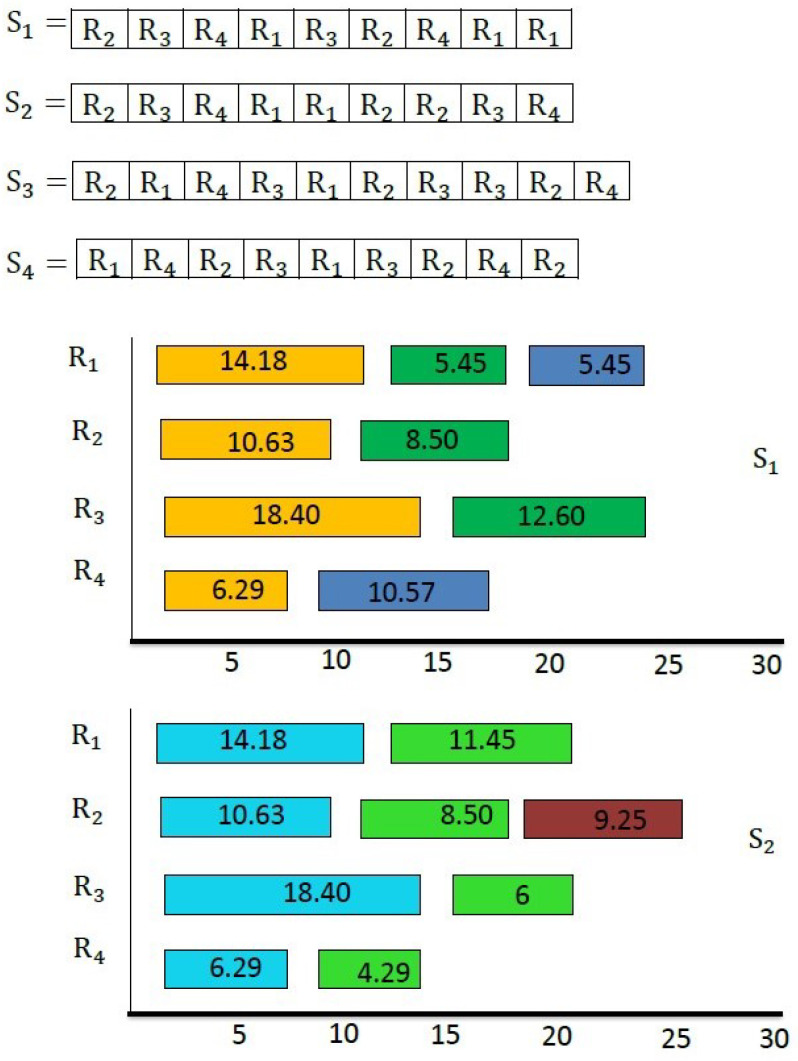
Corresponding Gantt chart of $S_{1}$ and $S_{2}$.

The greedy firefly algorithm starts with a random initial schedule, as shown in Figure 5. Figure 6 describes the corresponding schedule.

The greedy firefly algorithm generates a fitness value for each schedule in the second step. The fitness value represents the makespan time in the proposed greedy firefly algorithm. The makespan time is calculated using Equation (1).

(1) $$\begin{array}{c} makespantime=maxT_{1},T_{2},T_{3},\cdots ,T_{m} \end{array}$$

and

$$\begin{array}{c} T_{k}=\sum_{n}^{i=1}\frac{L_{i}}{S_{k}}, \end{array}$$

where $L_{i}$ represents the length of job${}_{i}$, and $S_{k}$ represents the speed of resource *k*.

$$T_{k}=\sum_{n}^{i=1}\frac{L_{i}}{S_{k}}=\begin{array}{ccccc} & S_{1} & S_{2} & S_{3} & S_{4}\\ R_{1} & 36.37 & 47.00 & 28.18 & 26.91\\ R_{2} & 19.13 & 28.38 & 22.88 & 18.50\\ R_{3} & 31.00 & 33.20 & 30.40 & 29.20\\ R_{4} & 13.11 & 10.57 & 10.57 & 17.43 \end{array}$$

$$Theinitailmakespantimes\;\left(\beta_{0}\right)=S_{1}=47.00,S_{2}=28.38,T_{3}=33.20,T_{4}=17.43$$

To calculate the brightness of each schedule, first we need to find the distances between each schedules using Equation (2) and (3):

(2) $$distance_{i,j}=∥x_{i}-x_{j}∥=\sqrt{\sum_{k=1}^{n}{\left(x_{i,k}-x_{j,k}\right)}^{2}}$$

(3) $$distanc_{i,j}=\left[\begin{array}{ccccc} \; & S_{1} & S_{2} & S_{3} & S_{4}\\ S_{1} & 0 & 2.00 & 3.46 & 5.10\\ S_{2} & 2.00 & 0 & 4.47 & 4.90\\ S_{3} & 3.46 & 4.47 & 0 & 5.10\\ S_{4} & 5.10 & 4.90 & 5.10 & 0 \end{array}\right]$$

The brightness of the schedule is calculated using Equation (4):

(4) $$\beta \left(r\right)=T_{k}e^{-\gamma distance_{i,j}^{2}}$$

The movements towards the brightest firefly is calculated using Equation (5):

(5) $$firefly_{i}\left(t+1\right)=firefly_{i}\left(t\right)+T_{k}e^{-\gamma distance_{i,j}^{2}}+\alpha \epsilon_{i}$$

The remaining steps of the proposed greedy firefly algorithm focus on the greedy choice by selected the schedule that handles as many jobs as possible on the cloud provider resources. Starting from the empty schedule, provided that at least one cloud job exists, continuously add in the job that leads to the minimum makespan time. The idea behind the proposed greedy firefly algorithm is to combine the benefits of firefly algorithm and greedy algorithms by using the greedy choice to help in enhancing the firefly algorithm schedule. The greedy choice allows the firefly solution to search for schedules near optimal solutions.

## 4. Performance Evaluation

Generally, the simple mathematical models of job scheduling metaheuristics fail when dynamic changes occur in the IoT grid configurations or the scheduling configuration. For this reason, the empirical evaluation of the greedy firefly algorithm is considered a good choice to assess the effectiveness of the proposed mechanism in the IoT grid. However, the empirical evaluation of the greedy firefly algorithm requires specifications of a wide range of parameters for the environment [65]. Therefore, simulation experiments have been conducted to assess the efficiency of the greedy firefly algorithm for IoT grid job scheduling. The simulation results focus on representing the performance advantages of the greedy firefly algorithm over the standard firefly algorithm and other states of arts scheduling mechanism.

GridSim simulator, a discrete event simulator, developed to simulate the distributed heterogeneous systems such as cloud systems and computational IoT grid environments, is used as a tool [66]. The workload traces to employed in our evaluation are obtained from the Grid Workload Achieve (GWA). Grid Workload Achieve is built based on a real grid workload trace as a public dataset to conduct real data experiments [67].

### 4.1. The Experiments

To evaluate the greedy firefly algorithm, the study considered different sizes of workload traces. As a result, the number of jobs range from lightweight loads with less than 1000 jobs to the heavy workload with 10,000 jobs. The parameter values for the proposed greedy firefly algorithm are described in Table 3 [43,64,68]. The experimental parameters in the study are based on the relevant literature [69,70,71,72].

Here, $\beta_{0}$ represents the attractiveness of each firefly at r=0, while γ represents the media light absorption coefficient, and α is a random number.

**Figure 7:**
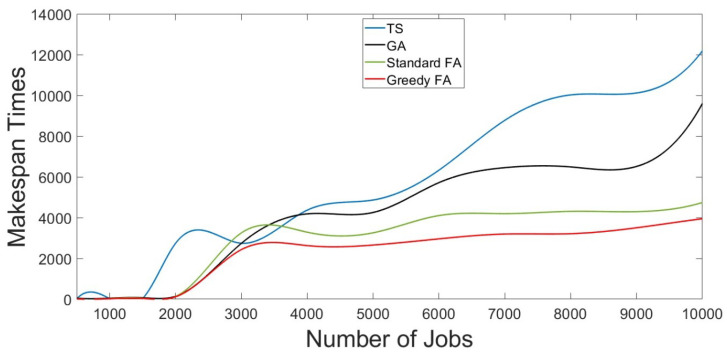
Makespan times of GFA Compared to Different Scheduling Methods for Different Workloads.

Table 4 and Figure 7 describes the makespan times of the greedy firefly algorithms compared to different IoT grid job scheduling methods for different workloads traces with sizes ranging from 500 jobs to 10,000 jobs.

Each scheduling method’s makespan and execution times were calculated to evaluate the proposed greedy firefly algorithm. The simulation results for the makespan and execution times are as stated in Table 4 and depicted in Figure 7. The results in Table 4 state that the proposed greedy firefly algorithm mostly has shorter makespan and execution times than other evaluated scheduling methods, such as TS, GA, and standard firefly algorithms. This means that the proposed greedy firefly algorithm needs a shorter time to finish the submitted jobs and has better performance than all other IoT grid job scheduling methods in different workload traces. Furthermore, the results show that the standard firefly algorithm is the second best. Therefore, these results prove that makespan times using the proposed greedy firefly algorithm have better performance than other evaluated IoT grid job scheduling algorithms.

The reasons for the improvements in the results demonstrate that the proposed GFA has better coverage as the search space increases. This is because the greedy algorithm increases the search exploitation process. The search exploitation implemented by the greedy algorithm increases the probability of finding the optimum solution. Furthermore, the study considers the balance between exploitation achieved by the greedy algorithm and the exploration implemented by the firefly algorithm.

**Figure 8:**
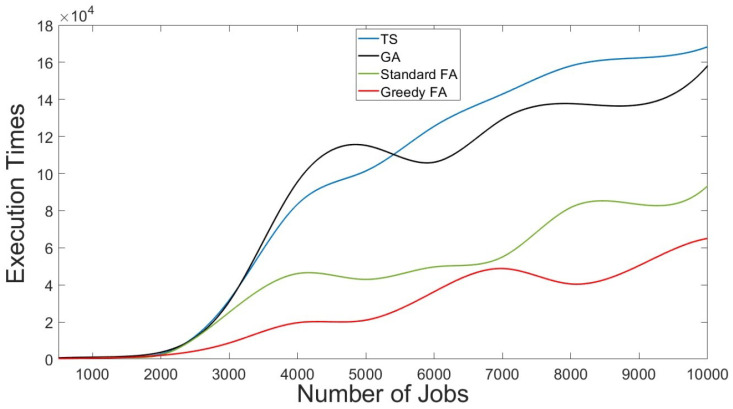
Execution times of the GFA Compared to Different Scheduling Methods for Different Workloads.

As reported in Table 5 and revealed in Figure 8 , the GFA has the shortest execution time compared to other evaluated scheduling methods. The obtained results indicate that proposed GFA improves the IoT grid job scheduling process when considering execution time as a performance metric.

### 4.2. Test Case 1: Typical Workload of 5000 Jobs

**Figure 9:**
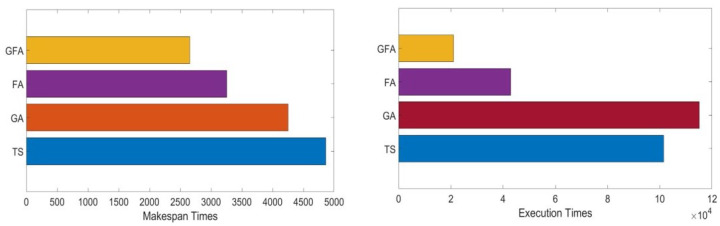
Makespan and Execution times for Typical Workloads.

To evaluate the efficiency of the GFA under different workloads, the study compared the makespan time and execution times of the proposed method with several scheduling algorithms using different workloads. The experiments evaluated the proposed GFA with a typical workload involving 5000 jobs in the first scenario. The makespan and execution times of varying scheduling methods are described in Table 6 and depicted in Figure 9.

Considering typical workload, the proposed GFA makespan time is 2654, the shortest makespan time compared with other evaluated scheduling algorithms in Table 6. Furthermore, the obtained results revealed that the GFA has the best execution time: 21,005 compared to 101,424, 115,148, and 4295 for TS, GA, and the standard firefly algorithm, respectively.

### 4.3. Test Case 2: The Heavy Load of 10,000 Jobs

**Figure 10:**
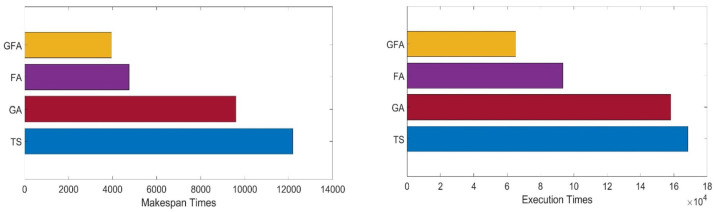
Makespan and Execution times for Heavy Workloads.

The study evaluated the GFA under a heavy workload environment containing 10,000 jobs. The makespan times and execution times of the TS, GA, and standard firefly algorithm and the GFA are reported in Table 7 and depicted in Figure 10.

**Figure 11:**
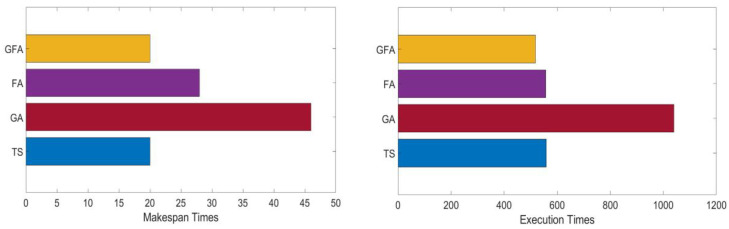
Makespan and Execution times for Lightweight Workloads.

Figure 10 and Table 7 compare the makespan time between the TS, GA, and standard firefly algorithm and the proposed GFA. These results show that the makespan time in the GFA is less than the other evaluated algorithms. In heavy workload, the proposed GFA produced a makespan time of 3945, which is shorter than the makespan time of 4742 for the standard firefly algorithm. As shown in Figure 11 and Table 8, the results obtained from this experiment show that the proposed GFA shows a shorter execution time compared to other evaluated scheduling methods. Moreover, GA is ranked far below the standard firefly algorithm in almost all test cases, and TS falls behind GA.

### 4.4. Test Case 3: Lightweight Load of 1000 Jobs

The study evaluated the GFA in a lightweight workload environment containing 1000 jobs. The makespan times and execution times of GFA and other evaluated scheduling methods are reported in Table 8 and depicted in Figure 11.

The makespan time for TS was 20 and for GA was 46, while the makespan time for the standard firefly algorithm was 28. The proposed GFA makespan time for this experiment was 20. As noted, the TS and GFA require shorter makespan times compared to other scheduling methods. The TS, GA, and standard firefly algorithms’ execution times were 559, 1040, and 557, respectively. Meanwhile, the execution time of the GFA was 518. These results prove that the proposed GFA is an effective method in optimizing the search performance for IoT grid scheduling problems since it minimizes the execution time required to obtain the optimal schedule. We also find that the standard firefly algorithm performs a little better than TS in lightweight workload cases; both fall behind the GFA. To conclude, the obtained results show that the GFA job scheduling mechanism can achieve better performance than the other scheduling mechanism. The GFA achieves better results in minimizing the makespan execution times of the IoT grid job scheduling process. Better GFA makespan and execution times may entice consumers to request GFA inorder to save money and complete their job on a reasonable time.

## 5. Conclusions

The performance of IoT grid environments depends mainly on the job scheduling method applied to manage resources. This paper presented an IoT grid job scheduling method based on a GFA. The proposed GFA depends on enhancing the efficiency of the standard firefly algorithm using the greedy approach. Furthermore, the proposed GFA aims to minimize the makespan and execution simultaneously. The study conducted various experiments to study the efficiency of the proposed GFA compared to those reported in recent work that used TS, GA, and standard firefly algorithms by conducting extensive simulation experiments using different workloads. The obtained simulation results revealed that the GFA has the shortest execution time, 21,005, compared to 101,424, 115,148, and 42,958 for the TS, GA, and standard firefly algorithm, respectively. Using the GFA for task scheduling, where the solution search space is huge, the GFA search takes a shorter time to find an optimal solution. This is because the greedy algorithm increases the search exploitation process. The search exploitation implemented by the greedy algorithm increases the probability of finding the optimum solution. Furthermore, the study considered the balance between exploitation achieved by the greedy algorithm and the exploration implemented by the firefly algorithm. Future research work for the GFA will include optimizing the process by integrating a clustering mechanism for clients’ tasks. Furthermore, the evaluation of the GFA will be extended based on other key parameters and considering a comparison with an additional scheduling strategy.

## Figures and Tables

**Table 1:** Data for the Example Instance Jobs.

Jobs	$J_{1}$	$J_{2}$	$J_{3}$	$J_{4}$	$J_{5}$	$J_{6}$	$J_{7}$	$J_{8}$	$J_{9}$
Length	85	92	44	78	63	68	74	30	30

**Table 2:** Data for the Example Instance Resources.

Resource	$R_{1}$	$R_{2}$	$R_{3}$	$R_{4}$
Speed	5.5	8	5	7

**Table 3:** Parameter values of Greedy Firefly Algorithm.

Parameters	Size of Population	α	γ	β	Number of iterations
Values	10	9	0.02	1.0	300

**Table 4:** Makespan times of The GFA Compared to Different Scheduling Methods for Different Workloads.

No. of Jobs	500	1000	1500	2000	3000	4000	5000	6000	7000	8000	9000	10,000
TS	25	20	30	2737	2743	4368	4865	6330	8776	10,022	10,118	12,184
GA	46	46	70	138	2739	4185	4253	5719	6452	6488	6512	9600
Firefly	18	28	46	129	3251	3284	3256	4102	4196	4307	4293	4742
GFA	12	20	32	114	2431	2627	2654	2958	3193	3211	3502	3945

**Table 5:** Execution times of the GFA Compared to Different Scheduling Methods for Different Workloads.

No. of Jobs	500	1000	1500	2000	3000	4000	5000	6000	7000	8000	9000	10,000
TS	25	20	30	2737	2743	4368	4865	6330	8776	10,022	10,118	12,184
GA	46	46	70	138	2739	4185	4253	5719	6452	6488	6512	9600
Firefly	18	28	46	129	3251	3284	3256	4102	4196	4307	4293	4742
GFA	12	20	32	114	2431	2627	2654	2958	3193	3211	3502	3945

**Table 6:** Makespan and Execution times for Typical Workloads.

No. of 5000 Jobs	TS	GA	Standard Firefly	GFA
GFA	4865	4253	3256	2654
Execution time	101,424	115,148	42,958	21,005

**Table 7:** Makespan and Execution times for Heavy Workloads.

No. of 5000 Jobs	TS	GA	Standard Firefly	GFA
Makespan Time	12,184	9600	4742	3945
Execution time	168,322	158,121	93,394	65,049

**Table 8:** Makespan and Execution times for Lightweight Workloads.

No. of 5000 Jobs	TS	GA	Standard Firefly	GFA
Makespan Time	20	46	28	20
Execution time	559	1040	557	518

## Data Availability

Not applicable.
